# A draft genome assembly of the solar-powered sea slug *Elysia chlorotica*

**DOI:** 10.1038/sdata.2019.22

**Published:** 2019-02-19

**Authors:** Huimin Cai, Qiye Li, Xiaodong Fang, Ji Li, Nicholas E. Curtis, Andreas Altenburger, Tomoko Shibata, Mingji Feng, Taro Maeda, Julie A. Schwartz, Shuji Shigenobu, Nina Lundholm, Tomoaki Nishiyama, Huanming Yang, Mitsuyasu Hasebe, Shuaicheng Li, Sidney K. Pierce, Jian Wang

**Affiliations:** 1 Department of Computer Science, City University of Hong Kong, Hong Kong 999077, China; 2 BGI-Shenzhen, Shenzhen 518083, China; 3 State Key Laboratory of Genetic Resources and Evolution, Kunming Institute of Zoology, Chinese Academy of Sciences, Kunming 650223, China; 4 Center for Excellence in Animal Evolution and Genetics, Chinese Academy of Sciences, 650223, Kunming, China; 5 BGI Genomics, BGI-Shenzhen, Shenzhen 518083, China; 6 Department of Biology, Ave Maria University, Ave Maria, Florida 34142, USA; 7 Section for Evolutionary Genomics, Natural History Museum of Denmark, University of Copenhagen, Copenhagen 1350, Denmark; 8 National Institute for Basic Biology, Okazaki 444-8585, Japan; 9 Department of Integrative Biology, University of South Florida, Tampa, Florida 33620, USA; 10 Graduate University for Advanced Studies (SOKENDAI), Okazaki 444-8585, Japan; 11 Advanced Science Research Center, Kanazawa University, Kanazawa 920-0934, Japan; 12 James D. Watson Institute of Genome Sciences, Hangzhou 310058, China; 13 Department of Biology, University of Maryland, College Park, Maryland 20742, USA

**Keywords:** Genome, DNA sequencing, Marine biology, Genomics, Evolution

## Abstract

*Elysia chlorotica*, a sacoglossan sea slug found off the East Coast of the United States, is well-known for its ability to sequester chloroplasts from its algal prey and survive by photosynthesis for up to 12 months in the absence of food supply. Here we present a draft genome assembly of *E. chlorotica* that was generated using a hybrid assembly strategy with Illumina short reads and PacBio long reads. The genome assembly comprised 9,989 scaffolds, with a total length of 557 Mb and a scaffold N50 of 442 kb. BUSCO assessment indicated that 93.3% of the expected metazoan genes were completely present in the genome assembly. Annotation of the *E. chlorotica* genome assembly identified 176 Mb (32.6%) of repetitive sequences and a total of 24,980 protein-coding genes. We anticipate that the annotated draft genome assembly of the *E. chlorotica* sea slug will promote the investigation of sacoglossan genetics, evolution, and particularly, the genetic signatures accounting for the long-term functioning of algal chloroplasts in an animal.

## Background & Summary

Many species of sacoglossan sea slugs are able to intracellularly sequester chloroplasts from their algal food, a phenomenon known as kleptoplasty, that is not observed in other clades of animals. In some sacoglossan species, the captured chloroplasts (usually called kleptoplasts) are maintained and capable of photosynthesis for one to several months, earning these molluscs the title of “solar-powered sea slugs”^1–3^. Among them, *Elysia chlorotica*, where the kleptoplasts are obtained from the filamentous alga *Vaucheria litorea*, is particularly interesting because it can retain functional chloroplasts in the cells of its digestive diverticula and survive without food supply for ten months to one year^2,3^. The mechanism that keeps ‘stolen’ chloroplasts functioning requires special proteins produced by nuclear genes of the algal host^4^. While there is a great deal of evidence using polymerase chain reaction (PCR)^5–10^, western blot^11,12^, RNA-seq^13^, and fluorescent in situ hybridization (FISH) investigations^14^ that algal nuclear genes are present in the sea slug, genomic resources are scarce for *E. chlorotica*, limited to a mitochondrial genome assembly^7^, a few transcriptomes^13,15,16^ and a low-coverage genome sequencing dataset of eggs^17^. There are no nuclear genome assemblies, even fragmented ones, publicly available for *E. chlorotica* so far. From an evolutionary perspective, although Mollusca represents the second largest animal phylum with around 85,000 extant species^18^, a fairly limited number of mollusc genomes have been sequenced yet^19–31^, with only 23 genomes publicly available on NCBI genome database (https://www.ncbi.nlm.nih.gov/genome/browse/#!/overview/mollusca; access on December 5, 2018). Particularly, no reference genome has been generated for any sacoglossan mollusc.

In this study, we present the first draft genome assembly for the representative solar-powered sea slug *E. chlorotica*, which was assembled from Illumina short and PacBio long reads using a hybrid and hierarchical assembly strategy. We anticipate that this well-annotated draft genome assembly and the massive sequencing data generated in this study will serve as substantial resources for future studies of the evolution of sacoglossan molluscs, and particularly, for the investigation of the genetic basis underlying the long-term maintenance of algal chloroplasts in these sea slugs.

## Methods

### Sample collection, library construction and sequencing

**Figure 1:**
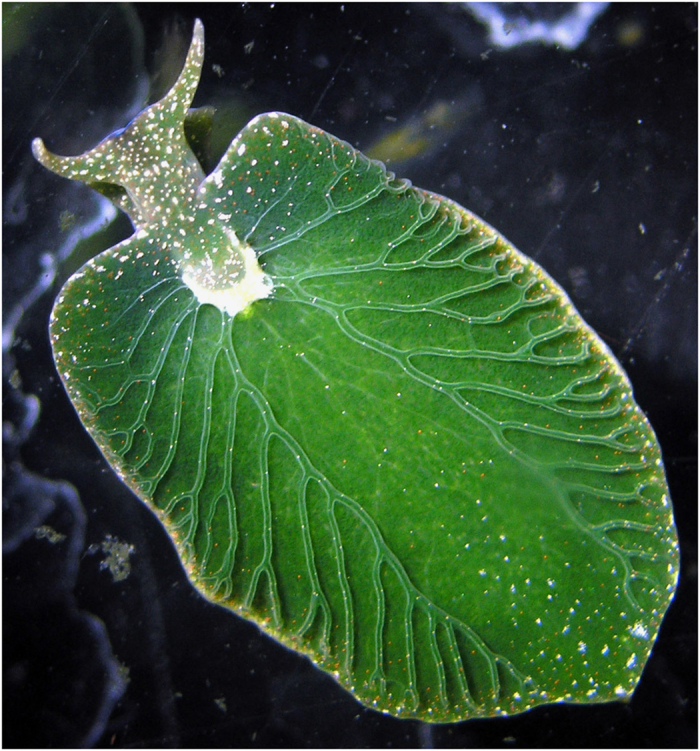
A photograph of an adult *Elysia chlorotica* (image courtesy of Patrick Krug).

Specimens of the sea slug *E. chlorotica* (NCBI taxonomy ID 188477; Fig. 1) were collected from a salt marsh near Menemsha on the island of Martha’s Vineyard, Massachusetts in 2010. From there, the animals were shipped to Tampa, Florida, for maintenance in aquaria containing sterile, artificial seawater (1000 mosm; Instant Ocean, VA, USA) on a 14:10 light–dark cycle at 10 °C as described in Pierce *et al.* (2012)^13^.

In Tampa, total DNA, including slug genomic, mitochondrial and algal chloroplast DNA, was extracted from a whole adult specimen that had been starved for at least 2 months using a Nucleon Phytopure DNA extraction kit (GE Healthcare UK limited, Buckinghamshire, UK) according to the manufacturer’s instructions. The same kit was used to extract total DNA, including slug genomic and mitochondrial DNA, from a batch of ~1000 larvae that had not hatched from the egg capsules and never been fed. The larvae do not have any chloroplasts. A total of 11 Illumina DNA paired-end (PE) libraries were constructed according to the standard protocol provided by Illumina (San Diego, CA, USA), including six libraries with short-insert sizes (170 bp × 2, 500 bp × 2, and 800 bp × 2) from the adult DNA, and five mate-paired libraries with long-insert sizes (2.5 kb × 2, 5 kb × 2, 10 kb × 1) from the larval DNA. Sequencing was performed for all the 11 libraries on the HiSeq 2000 platform according to the manufacturer’s instructions (Illumina, San Diego, CA, USA), using the modes of PE100 for all the short-insert libraries and PE49 + PE90 for each of the five mate-paired libraries. A total of 296.73 Gb of Illumina reads were produced (Data Citation 1 and Data Citation 2), which can cover the estimated haploid genome size of *E. chlorotica* by *k*-mer analysis for 516 times (Table 1).

In addition, 9.45 Gb (16X) PacBio long-reads with a mean subread length of 1.2 kb and N50 subread length of 1.7 kb were sequenced for another DNA sample (Data Citation 1 and Data Citation 2), which was extracted from another starved adult specimen, that had been shipped frozen to Okazaki, using the CTAB method^32^ and purified with DNeasy Plant mini kit (Qiagen). Three libraries were constructed according to the 6 kb library construction protocol and sequenced in 93 SMRT cells on the PacBio RS platform using the C2 chemistry following the manufacturer’s instructions (Pacific Biosciences, Menlo Park, CA, USA).

### Estimation of genome size and heterozygosity

Prior to downstream analyses, all the Illumina reads were submitted to strict quality control using SOAPnuke (v1.5.3)^33^. Duplicated reads arising from PCR amplification during library construction, adapter-contaminated reads and low-quality reads were removed using parameters *-l 7 -q 0.4 -n 0.02 -d -t 10,0,10,0* for the short-insert (i.e. 170 bp, 500 bp and 800 bp) data and *-l 7 -q 0.35 -n 0.05 -d -S* for the long-insert (i.e. 2.5 kb, 5 kb and 10 kb) data, yielding a total of 176.32 Gb of clean Illumina reads (Table 1).

All of the clean reads from the six short-insert libraries, except those derived from algal chloroplasts, slug mitochondria and the previously reported endogenous retrovirus of *E. chlorotica*^34^, were used to estimate the size and heterozygosity of the *E. chlorotica* nuclear genome by *k*-mer analysis. Reads were considered to be derived from organelles or retrovirus if a pair of reads was mapped to any of the three genomes (Data Citation 3, 4, 5) by BWA-MEM (v0.7.16)^35^, and such read pairs were discarded, resulting in a total of 62.8 Gb Illumina data for *k*-mer analysis. The haploid genome size of *E. chlorotica* was estimated to be around 575 Mb according to *k*-mer frequency distributions generated by Jellyfish (v2.2.6)^36^ using a series of *k* values (17, 19, 21, 23, 25, 27, 29 and 31) with the *-C* setting, which was calculated as the number of effective *k*-mers (i.e. total *k*-mers – erroneous *k*-mers) divided by the homozygous peak depth (Table 2). But this estimated haploid genome size might be an underestimate, as certain parts of the *E. chlorotica* genome (e.g. GC-extreme regions) may have failed to be sequenced due to technical limitations^37^, and/or repetitive sequences may not have been resolved properly by *k*-mer analysis given that mollusc genomes are generally known to be repeat rich^19–31^.

**Figure 2:**
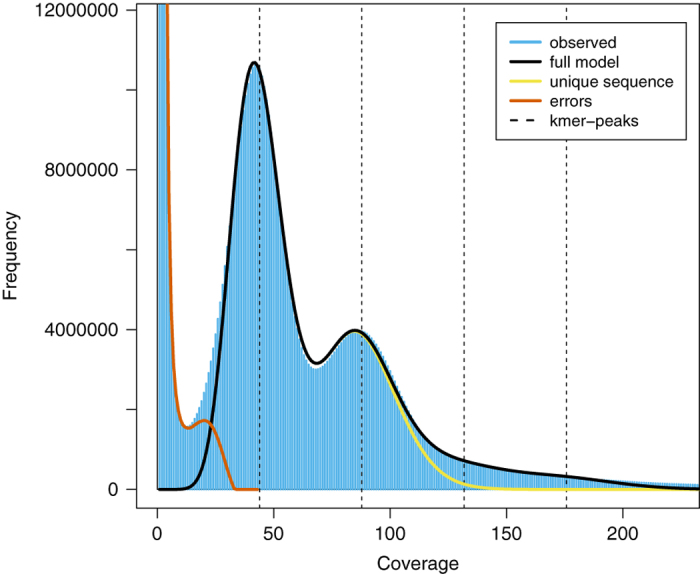
A 17-mer frequency distribution of *E. chlorotica* based on 62.8 Gb Illumina data. The first peak at coverage 43X corresponds to the heterozygous peak. The second peak at coverage 86X corresponds to the homozygous peak.

A double-peak *k*-mer distribution with the heterozygous peak (1^st^ peak) being much higher than the homozygous peak (2^nd^ peak) strongly indicates that *E. chlorotica* has a diploid genome with a high level of heterozygosity (Fig. 2). The rate of heterozygosity was estimated to be around 3.66% by GenomeScope (v1.0.0)^38^ with the *k*-mer frequency distributions generated by Jellyfish as inputs (Table 2). The heterozygosity rate of *E. chlorotica* (3.66%) was higher than those rates in the bivalve molluscs [*Bathymodiolus platifrons* (1.24%)^19^, *Modiolus philippinarum* (2.02%)^19^, *Limnoperna fortune* (2.3%)^23^, *Pinctada fucata martensii* (2.5–3%)^27^ and *Chlamys farreri* (1.4%)^39^] and a freshwater shelled gastropod [*Pomacea canaliculata* (1–2%)^30^], all also estimated by *k*-mer analyses, highlighting the difficulty of assembling the *E. chlorotica* genome.

### Genome assembly

The *E. chlorotica* genome was assembled by a hybrid and hierarchical assembly strategy as described below: (i) Clean reads from the Illumina short- and long-insert libraries were assembled into contigs using ALLPATHS-LG (v52488)^40^ with default parameters except setting *HAPLOIDIFY = True*, which yielded an initial assembly with a total length of 776 Mb and a contig N50 of 1.7 kb. This initial assembly was ~35% longer than the estimated genome size of 575 Mb, indicating that, for some genomic regions, two haploids were assembled separately due to high heterozygosity. Thus, (ii) we used HaploMerger2 (v20151124)^41^ to separate the two haploid sub-assemblies from the initial ALLPATHS-LG assembly, and an assembly with a total length of 575 Mb and contig N50 of 1.9 kb was produced. Next, (iii) we assembled a separate genome with the PacBio long-reads alone. Sequencing errors in the PacBio reads were first corrected by the clean Illumina reads from 170 bp and 500 bp short-insert libraries using PacBioToCA (v8.3)^42^ with parameter *-length 400*, and 5.36 Gb (9.32 X) error-corrected PacBio reads were retained (Table 1). Then the error-corrected PacBio reads were assembled using Canu (v1.4)^43^ with parameters *minReadLength = 400 minOverlapLength = 400 contigFilter = 2 400 1.0 1.0 2*, which produced an assembly with a total length of 469 Mb and contig N50 of 4.4 kb. (iv) The PacBio assembly was merged with the above HaploMerger2 assembly with Metassembler (v1.5)^44^, which resulted in an improved assembly with a total length of 535 Mb and contig N50 of 5.1 kb. (v) These resulting contigs were further assembled into scaffolds using the distance information provided by read pairs from the Illumina short- and long-insert libraries with SSPACE (STANDARD-3.0)^45^. Specifically, prior to scaffolding, the read pairs were aligned to the contigs using BWA (v0.6.2), and the insert size of each library was inferred from the statistics of a pre-run of SSPACE based on satisfied pairs in distance and orientation within contigs. Then scaffolding was performed with SSPACE using the estimated insert size of each library with the minimum allowed insert size error setting to be 0.3 for short-insert libraries and 0.5 for long-insert libraries. Subsequently, (vi) intra-scaffold gaps were filled using PBJelly from PBSuite (v15.8.24)^46,47^ with the error-corrected PacBio long reads by setting *minReads = 3*, followed by using GapCloser (v1.10.1)^48^ with the Illumina short-insert paired-end reads by setting library insert sizes according to SSPACE estimation as described above. (vii) The gap-filled scaffolds were submitted to HaploMerger2 again to reduce redundant sequences, followed by polishing with all the Illumina short-insert clean reads by PILON (v1.22)^49^. Finally, (viii) potential contaminants in the assembly including sequences from algal chloroplasts, slug mitochondria and adaptor/vector as identified by the NCBI contamination-screening pipeline were removed by an in-house script. The improvements of assembly generated at each step of the assembly process were presented in Table 3.

The final result was a genome assembly with a total length of 557 Mb, comprising 9,989 scaffolds (Data Citation 6,7). The contig and scaffold N50s of this assembly were 28.5 kb and 442.0 kb, respectively, and unclosed gap regions represented 3% of the assembly (Table 4), exhibiting a continuity comparable to other published molluscan genomes (Data Citation 8, 9, 10, 11, 12, 13, 14, 15, 16, 17, 18, 19, 20, 21). In addition, GC content of the *E. chlorotica* assembly excluding gaps was estimated to be 37.7%.

### Repetitive element annotation

Repetitive elements in the *E. chlorotica* genome assembly were identified by homology searches against known repeat databases and *de novo* predictions. Briefly, we carried out homology searches for known repetitive elements in the *E. chlorotica* assembly by screening the Repbase-derived RepeatMasker libraries (v20170127) with RepeatMasker (v4.0.7; setting *-nolow -norna -no_is*)^50^ and the transposable element protein database with RepeatProteinMask (an application within the RepeatMasker package; setting *-noLowSimple -pvalue 0.0001 -engine ncbi*). For *de novo* prediction, RepeatModeler (v1.0.11)^51^ was executed on the *E. chlorotica* assembly to build a *de novo* repeat library for *E. chlorotica*. Then RepeatMasker was employed to align sequences from the *E. chlorotica* assembly to the *de novo* library for identifying repetitive elements. We also searched the genome assembly for tandem repeats using Tandem Repeats Finder (v4.09)^52^ with parameters *Match = 2 Mismatch = 7 Delta = 7 PM = 80 PI = 10 Minscore = 50 MaxPeriod = 2000*. Overall, we identified 176 Mb of non-redundant repetitive sequences, representing 32.6% of the *E. chlorotica* genome assembly excluding gaps (Table 5). Of note, the *E. chlorotica* repeat repertoire is highly diverse, comprising 33.5 Mb of DNA transposons (6.2% of the assembly), 30.3 Mb of long interspersed elements (LINEs; 5.6%), 19.4 Mb of short interspersed nuclear elements (SINEs; 3.6%), 14.4 Mb of long terminal repeats (LTRs; 2.7%), and 55.8 Mb of tandem repeats (10.3%; Table 5).

### Protein-coding gene annotation

We applied a combination of homology-based, transcriptome-based and *de novo* prediction methods to build consensus gene models for the *E. chlorotica* genome assembly. For homology-based prediction, protein sequences of *Aplysia californica*, *Caenorhabditis elegans*, *Crassostrea gigas*, *Drosophila melanogaster*, *Lottia gigantea* and *Homo sapiens* were first aligned to the *E. chlorotica* assembly using TBLASTN (blast-2.2.26)^53^ with parameters *-F F -e 1e-5*. Then the genomic sequences of the candidate loci together with 2 kb flanking sequences were extracted and submitted to GeneWise (wise-2.4.1)^54^ for exon-intron structure determination by aligning the homologous proteins to these extracted genomic sequences with settings of *-sum -genesf -gff -tfor/-trev* (*-tfor* for genes on forward strand and *-trev* for reverse strand). For transcriptome-based prediction, we collected published RNA-seq data from Pierce *et al.* (2012)^13^ (Data Citation 22) and Chan *et al.* (2018)^15^ (Data Citation 23), representing a total of 32.3 Gb of RNA reads from 13 samples across different developmental stages of *E. chlorotica* (from juvenile to adult) upon exposure to the algal food *V. litorea*. All the RNA-seq reads were first submitted to SOAPnuke (v1.5.6) for quality control by removal of adapter-contaminated reads and low-quality reads with parameters *-Q 1 -G -t 15,0,15,0 -l 20 -q 0.2 -E 60 -5 1* for paired-end data from Pierce *et al.* (2012) and *-Q 2 -G -t 15,0,0,0 -l 20 -q 0.2 -E 60 -5 1* for single-end data from Chan *et al.* (2018). We then mapped the clean RNA reads to the *E. chlorotica* genome using HISAT2 (v2.1.0)^55^ and assembled transcripts by StringTie (v1.3.3b)^56^. For *de novo* prediction, we first randomly picked 800 transcriptome-based gene models with complete open reading frames (ORFs) and reciprocal aligning rates exceeding 80% against homologous proteins in the UniProtKB/Swiss-Prot database (v2018_05)^57^ to train AUGUSTUS (v3.3.1)^58^ in order to obtain parameters suitable for *E. chlorotica* genes. Then we performed *de novo* prediction on the repeat-masked genome using AUGUSTUS with the obtained gene parameters and *--uniqueGeneId = true --noInFrameStop = true --gff3 = on*.

Finally, gene models from the above three methods were combined into a non-redundant gene set using a similar strategy as Xiong *et al.* (2016)^59^. Briefly, the homology-based gene models were first integrated with the transcriptome-based models to form a core gene set, followed by integration with the *de novo* models. Then *de novo* models not supported by homology-based and transcriptome-based evidence were also added to the core gene set if BLASTP (blast-2.2.26; parameters *-F F -e 1e-5*)^53^ hits could be found in the UniProtKB/Swiss-Prot database (v2018_05). Finally, genes showing BLASTP (blast-2.2.26; parameters *-F F -e 1e-5*) hits to transposon proteins in the UniProtKB/Swiss-Prot database (v2018_05) were removed from the combined gene set. We ultimately obtained 24,980 protein-coding genes with up to 22,717 (90.9%) supported by RNA-seq signal ( ≥ 5 RNA reads).

To assign gene names for each predicted protein-coding locus, we first mapped the protein sequences of all the 24,980 genes to the UniProtKB/Swiss-Prot database (v2018_05) using BLASTP (blast-2.2.26)^53^ with parameters *-F F -e 1e-5*. Then the best hit of each gene was retained based on its BLASTP bit score, and the gene name of this best hit was assigned to the query *E. chlorotica* gene. A similar process was performed against the NCBI nr database (v20180315). In addition, we performed functional annotation with InterProScan (v5.29-68.0)^60^ to examine motifs, domains, and other signatures by searching against the databases including ProDom^61^, PRINTS^62^, Pfam^63^, SMART^64^, PANTHER^65^ and PROSITE^66^. To determine what pathways the *E. chlorotica* genes might be involved in, protein sequences of the *E. chlorotica* genes were searched against the KEGG database (v87)^67^ with BLASTP (blast-2.2.26) using parameters *-F F -e 1e-5*. As a result, 21,452 (85.9%) of the predicted protein-coding genes were successfully annotated by at least one of the four methods (Table 6).

### Code availability

The bioinformatic tools used in this work, including versions, settings and parameters, have been described in the Methods section. Default parameters were applied if no parameters were mentioned for a tool.

## Data Records

Raw reads from Illumina and PacBio sequencing are deposited in the NCBI Sequence Read Archive (SRA) database with accession number SRP156455 and Bioproject accession PRJNA484060 (Data Citation 1) and are also deposited in the CNGB Nucleotide Sequence Archive (CNSA) with accession number CNP0000110 (https://db.cngb.org/cnsa; Data Citation 2). Genome assembly, gene and repeat annotation of *E. chlorotica* generated in this study are deposited in the figshare repository (Data Citation 6) and NCBI under accession number GCA_003991915.1 (Data Citation 7).

## Technical Validation

**Figure 3:**
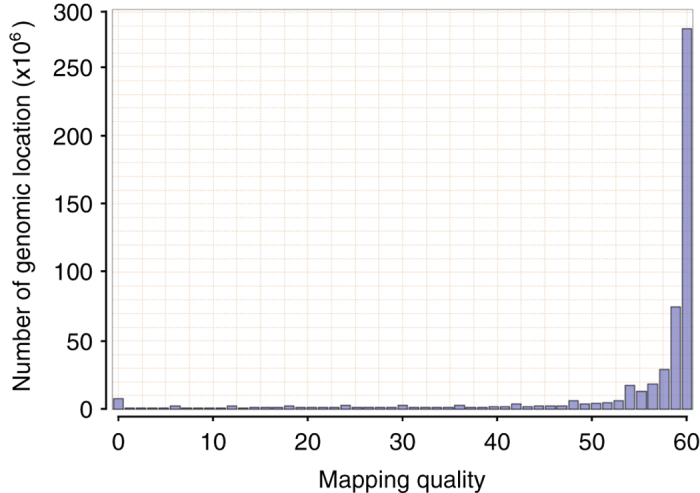
Mapping quality distribution of the *E. chlorotica* genome assembly. The distribution was generated by Qualimap 2 (v2.2.1) with the BWA-MEM (v0.7.16) alignment of 62.8 Gb short-insert Illumina clean data as input.

**Figure 4:**
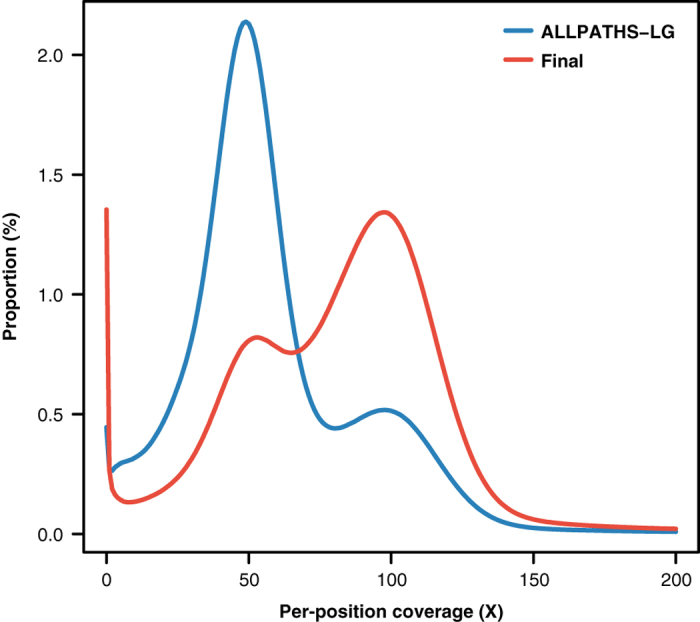
Per-position coverage distributions of the initial ALLPATHS-LG assembly and the final genome assembly. Per-position coverage was counted based on the BWA-MEM (v0.7.16) alignment of 62.8 Gb short-insert Illumina clean data with PCR duplicates removed by Picard (v2.10.10).

**Figure 5:**
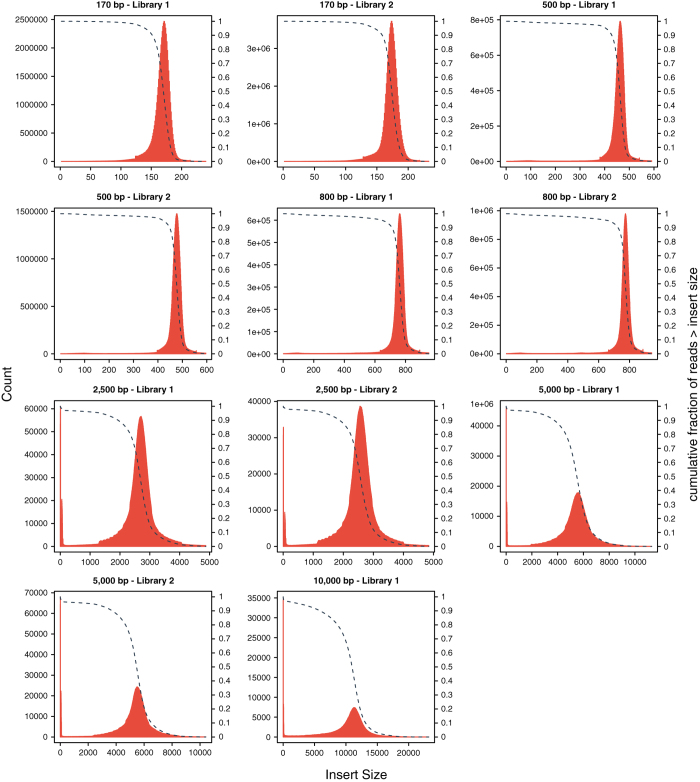
Fragment size distributions for all the Illumina libraries. The distributions were generated by Picard CollectInsertSizeMetrics (v2.10.10; setting *MINIMUM_PCT = 0.5*) with BWA-MEM (v0.7.16) alignment of read pairs from each library as input.

To evaluate the quality of the *E. chlorotica* assembly, we first aligned the 62.8 Gb Illumina short-insert clean reads which were previously used for *k*-mer analysis to the assembly using BWA-MEM (v0.7.16)^35^, and observed that 96% could be mapped back to the assembled genome with 85% of the mapped reads being aligned in proper pairs as counted by samtools flagstat (SAMtools v1.7)^68^. Qualimap 2 (v2.2.1)^69^ analysis reported a mean mapping quality (MQ) of 50.36 across all genomic positions in the final assembly with most positions having MQ of 60 (i.e. the highest MQ of BWA-MEM; Fig. 3). Moreover, single-position coverage analysis by samtools bedcov based on the above BWA-MEM alignment with PCR duplicates removed by Picard (v2.10.10)^70^ revealed that 98% of the assembly excluding gaps were covered by ≥ 5 reads and resulted in a per-position coverage distribution with its peak at 98X (Fig. 4). Of note, a minor peak at 49X was also observed (Fig. 4), implying that either a small amount of redundant sequences was still present in the final assembly, or the genomic regions corresponding to the minor peak were highly polymorphic between two haploids so that reads from the unassembled allele could not be aligned to the assembled allele by BWA-MEM due to low sequence identity. We also used Picard CollectInsertSizeMetrics (v2.10.10; setting *MINIMUM_PCT = 0.5*) to analyze the insert size distribution of each Illumina library based on BWA-MEM (v0.7.16) alignment of read pairs from each library, and observed that the estimated insert sizes of all the libraries matched their expected fragment sizes (Fig. 5). These results indicated that most sequences of the *E. chlorotica* genome that were captured by the sequencing platforms are present in the current assembly with proper orientation.

**Figure 6:**
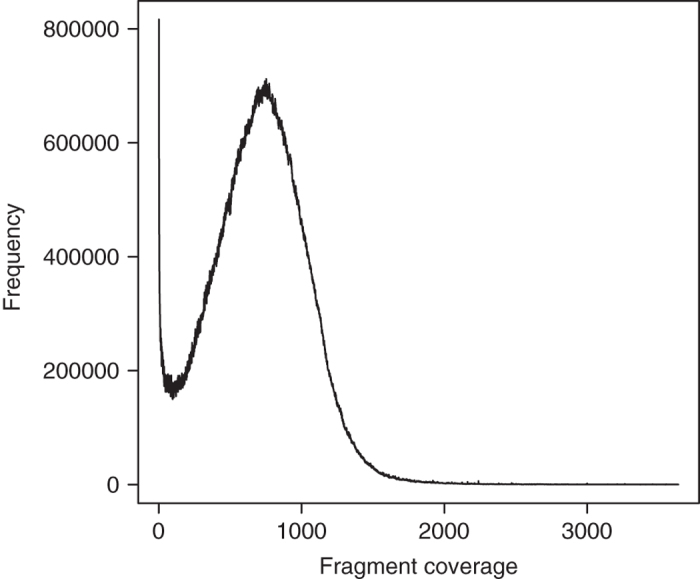
Fragment coverage distribution of the 10 kb mate-pair library data generated by REAPR (v1.0.18).

The quality of the *E. chlorotica* assembly was further assessed by REAPR (v1.0.18)^71^, a tool that evaluates the accuracy of a genome assembly using mapped paired-end reads. Specifically, all the short-insert clean reads and the 10 kb mate-pair reads were aligned to the final assembly by reapr smaltmap for calling error-free bases and scaffolding errors, respectively. It is noteworthy that REAPR recommends using the longest insert data with sufficient fragment coverage for calling scaffolding errors while data from multiple long-insert libraries are available^71^. In our case, the fragment coverage of the 10 kb mate-pair library calculated by REAPR peaked at 752X (Fig. 6), far beyond the minimum requirement of 15X. Ultimately, REAPR judged 80.45% of the bases in the *E. chlorotica* assembly as error free (i.e. bases covered by ≥ 5 perfectly and uniquely mapped reads), and identified 123 collapsed repeats and a total of 2,943 fragment coverage distribution (FCD) errors. An FCD error usually represents incorrect scaffolding, a large insertion or deletion in the assembly^71^. Considering the high heterozygosity (3.66%), the high repeat content (32.6%) and especially the high tandem repeat content (10.3%) of this genome, which likely affect the performance of read mapping, we believe that the accuracy of the *E. chlorotica* assembly is acceptable. As a comparison, more than 7,000 FCD errors are recently reported in an improved genome assembly of the Atlantic cod *Gadus morhua*, of which the assembly size (643 Mb) and the tandem repeat content (11% of assembly) are actually comparable to the *E. chlorotica* assembly^72^.

Next, we employed Benchmarking Universal Single-Copy Orthologs (BUSCO, v3.0.2)^73^ , a software package that can quantitatively measure genome assembly completeness based on evolutionarily informed expectations of gene content, to evaluate the completeness of the *E. chlorotica* assembly and 14 other published molluscan genomes using 978 genes that are expected to be present in all metazoans. We found that 927 (94.7%) of the expected genes were present in the *E. chlorotica* assembly with 913 (93.3%) and 14 (1.5%) identified as complete and fragmented, respectively. Only 51 (5.3%) genes were considered missing in the *E. chlorotica* assembly. The completeness of the *E. chlorotica* assembly based on BUSCO assessment was overall comparable to other published molluscan genome assemblies (Table 4).

Finally, we evaluated the completeness of the annotated gene set of *E. chlorotica* with BUSCO (v3.0.2) and DOGMA (v3.0)^74^, a program that measures the completeness of a given transcriptome or proteome based on a core set of conserved domain arrangements (CDAs). BUSCO analysis based on the metazoan dataset showed that 968 (98.9%) of the expected genes were present in the *E. chlorotica* gene set with 948 (96.9%) identified as complete. A higher number of expected genes were identified by BUSCO in the annotated gene set than in the *E. chlorotica* genome assembly, probably because searching genes in a transcriptome or proteome is simpler than in a genome. Meanwhile, DOGMA analysis based on PfamScan Annotations (PfamScan v1.5; Pfam v32.0)^63^ and the eukaryotic core set identified 93.3% of the expected CDAs in the annotated gene set. These results demonstrate the completeness of the annotated gene set of the *E. chlorotica* assembly.

## Additional information

**How to cite this article**: Cai, H. *et al*. A draft genome assembly of the solar-powered sea slug *Elysia chlorotica*. *Sci. Data*. 6:190022 https://doi.org/10.1038/sdata.2019.22 (2019).

**Publisher’s note**: Springer Nature remains neutral with regard to jurisdictional claims in published maps and institutional affiliations.

## Supplementary Material



## Figures and Tables

**Table 1:** Statistics of DNA reads produced for the *E. chlorotica* genome in this study.

Platform	Insert size (bp)	No. of Libraries	Read length (bp)	Raw data										Clean data		
				Total bases (Gb)	Sequencing coverage (X)	Physical coverage (X)	Total bases (Gb)	Sequencing coverage (X)	Physical coverage (X)							
Illumina	170	2	100	35.55	61.83	52.55	28.67	49.86	41.56
	500	2	100	32.44	56.42	141.06	18.12	31.51	84.04
	800	2	100	34.51	60.02	240.04	21.01	36.54	158.32
	2,500	2	49;90	76.60	133.22	1,752.62	43.77	76.12	1,301.05
	5,000	2	49;90	83.67	145.51	4,771.57	46.30	80.52	2,767.88
	10,000	1	49;90	33.97	59.08	3,841.34	18.45	32.09	2,174.84
	Total	11	—	296.73	516.05	10,799.18	176.32	306.64	6,527.68
PacBio	6,000	3	1,224	9.45	16.43	—	5.36	9.32	—
Note: Each of the five Illumina mate-pair libraries (2.5 kb × 2, 5 kb × 2 and 10 kb × 1) was run on two lanes with read length of 49 bp and 90 bp, respectively. Coverage calculation was based on the estimated genome size of 575 Mb according to *k*-mer analysis. Sequence coverage is the average number of times a base is read, while physical coverage is the average number of times a base is spanned by sequenced fragments.									

**Table 2:** Estimation of genome size and heterozygosity of *E. chlorotica* by *k*-mer analysis.

*k*	Total number of *k*-mers	Minimum coverage (X)	Number of erroneous *k*-mers	Homozygous peak	Estimated genome size (Mb)	Estimated heterozygosity (%)
17	51,187,863,592	13	1,410,585,877	86	579	3.59
19	49,735,232,800	11	1,804,614,643	84	571	3.93
21	48,282,601,880	11	2,010,206,114	80	578	3.90
23	46,829,970,960	11	2,152,586,758	78	573	3.79
25	45,377,340,040	10	2,235,304,391	75	575	3.69
27	43,924,709,120	10	2,327,591,479	72	578	3.57
29	42,472,078,200	9	2,370,847,012	70	573	3.47
31	41,019,447,280	9	2,433,307,151	67	576	3.36
Note: *k*-mer frequency distributions were generated by Jellyfish (v2.2.6) using 62.8 Gb Illumina clean data as input and a series of *k* values (17, 19, 21, 23, 25, 27, 29 and 31) with the -C setting. Minimum coverage was the coverage depth value of the first trough in *k*-mer frequency distribution. *k*-mers with coverage depth less than the minimum coverage were regarded as erroneous *k*-mers. Estimated genome size was calculated as (Total number of *k*-mers – Number of erroneous *k*-mers)/Homozygous peak.						

**Table 3:** Improvement in continuity and completeness of genome assembly generated by each of the eight assembly steps as stated in main text.

Step	Assembly statistics										Read mapping assessment		BUSCO assessment		
	Assembly size (Mb)	Contig N50 (kb)	Scaffold N50 (kb)	Gap ratio (%)	Mapping rate (%)	Mapping rate in proper pairs (%)	Complete BUSCOs (%)	Fragmented BUSCOs (%)	Missing BUSCOs (%)						
i	776	1.7	NA	0	95.27	65.07	30.2	37.5	32.3
ii	575	1.9	NA	0	93.26	63.91	29.2	38.9	31.9
iii	469	4.4	NA	0	81.42	79.45	54.5	26.6	18.9
iv	535	5.1	NA	0	95.20	79.79	64.9	25.3	9.8
v	583	5.6	457.2	8.27	95.35	82.77	92.0	2.0	6.0
vi	584	27.6	455.6	3.03	96.39	84.12	92.8	1.6	5.6
vii	560	28.5	457.0	3.03	96.06	83.89	93.2	1.5	5.3
viii	557	28.5	442.0	3.04	95.93	83.87	93.3	1.4	5.3
Note: For read mapping assessment, 500,000 pairs of clean reads were randomly selected from each of the six short-insert libraries, summed up to 3 M pairs of clean reads, which were aligned to each assembly by BWA-MEM (v0.7.16), followed by mapping rates counting by samtools flagstat (SAMtools v1.7). For BUSCO assessment, the percentages of complete, fragmented and missing BUSCOs were calculated by BUSCO (v3.0.2) for all the assemblies using 978 genes that are expected to be present in all metazoans.									

**Table 4:** Comparison of assembly continuity and completeness for available mollusc genomes.

Species	Sequencing technology	Genome coverage (X)	Assembly size (Mb)	Contig N50 (kb)	Scaffold N50 (kb)	Gap ratio (%)	Complete BUSCOs (%)	Fragmented BUSCOs (%)	Assembly Data Citation
*Aplysia californica*	Illumina	66	927.31	9.59	917.54	20.44	92.5	2.0	8
*Bathymodiolus platifrons*^19^	Illumina	319	1,658.19	10.74	343.34	11.77	93.6	2.5	9
*Biomphalaria glabrata*^20^	454	28	916.39	7.30	48.06	1.91	88.9	4.9	10
*Crassostrea gigas*^21^	Illumina + Fosmid	100	557.74	31.24	401.69	11.81	95.2	1.1	11
*Haliotis discus hannai*^22^	Illumina + PacBio	322	1,865.48	14.19	200.10	6.25	91.6	4.9	12
*Limnoperna fortunei*^23^	Illumina + PacBio	60	1,673.22	32.17	309.12	0.23	81.9	7.3	13
*Lottia gigantea*^24^	Sanger	9	359.51	93.95	1,870.06	16.86	95.9	0.9	14
*Modiolus philippinarum*^19^	Illumina	209	2,629.56	13.66	100.16	4.84	89.8	5.0	15
*Octopus bimaculoides*^25^	Illumina	92	2,338.19	5.53	475.18	15.13	90.4	3.6	16
*Patinopecten yessoensis*^26^	Illumina	297	987.59	37.58	803.63	8.10	94.3	1.3	17
*Pinctada fucata martensii*^27^	Illumina + BACs + RAD-seq	150	990.98	21.52	59,032.46	11.18	87.8	3.5	18
*Pomacea canaliculata*^30^	Illumina + PacBio + Hi-C	60	440.16	1072.86	31,531.29	0.02	95.8	0.7	19
*Radix auricularia*^28^	Illumina	72	909.76	16.26	578.73	6.42	93.2	1.5	20
*Saccostrea glomerata*^31^	Illumina	300	788.10	39.54	804.23	5.27	91.9	3.8	21
*Elysia chlorotica*	Illumina + PacBio	316	557.48	28.55	441.95	3.04	93.3	1.4	6,7
Note: Sequencing technology and genome coverage were retrieved from the indicated reference or data citation for each species. Assembly size, Contig N50, Scaffold N50 and Gap ratio were calculated with an in-house script according to assemblies downloaded from NCBI or GigaDB with indicated Data Citations. The percentages of complete and fragmented BUSCOs were calculated by BUSCO (v3.0.2) for the all the assemblies using 978 genes that are expected to be present in all metazoans.									

**Table 5:** Statistics for repetitive sequences identified in the *E. chlorotica* genome assembly according to detection method and biological category.

According to method			According to category		
Tool	Total repeat length (bp)	% of assembly	Category	Total repeat length (bp)	% of assembly
RepeatMasker	51,434,719	9.52	DNA	33,515,133	6.20
RepeatProteinMask	12,318,674	2.28	LINE	30,286,412	5.60
RepeatModeler	127,879,238	23.66	SINE	19,423,541	3.59
Tandem Repeats Finder	55,758,776	10.32	LTR	14,375,566	2.66
Combined	176,039,101	32.57	Tandem repeats	55,758,776	10.32

**Table 6:** Summary of protein-coding gene annotation for the *E. chlorotica* genome assembly.

Total number of protein-coding genes	24,980
Gene space (exon + intron; Mb)	233.5 (41.9% of assembly)
Mean gene size (bp)	9,634
Mean CDS length (bp)	1,344
Exon space (Mb)	33.2 (6.0% of assembly)
Mean exon number per gene	6.8
Mean exon length (bp)	198
Mean intron length (bp)	1,433
% of proteins with hits in UniProtKB/Swiss-Prot	61.3
% of proteins with hits in NCBI nr database	84.1
% of proteins with signatures assigned by InterProScan	68.8
% of proteins with KO assigned by KEGG	64.7
% of proteins with functional annotation	85.9
